# Effects of Neural Adaptation to Habitual Spherical Aberration on Depth of Focus

**DOI:** 10.21203/rs.3.rs-3917931/v1

**Published:** 2024-02-16

**Authors:** Seung Pil Bang, Ramkumar Sabesan, Geunyoung Yoon

**Affiliations:** University of Rochester; University of Washington School of Medicine; University of Houston

## Abstract

We investigated how long-term visual experience with habitual spherical aberration (SA) influences subjective depth of focus (DoF). Nine healthy cycloplegic eyes with habitual SAs of different signs and magnitudes were enrolled. An adaptive optics (AO) visual simulator was used to measure through-focus high-contrast visual acuity after correcting all monochromatic aberrations and imposing +0.5 μm and −0.5 μm SAs for a 6-mm pupil. The positive (n=6) and negative (n=3) SA groups ranged from 0.17 to 0.8 μm and from −1.2 to −0.12 μm for a 6-mm pupil, respectively. For the positive habitual SA group, the median DoF with positive AO-induced SA (2.18D) was larger than that with negative AO-induced SA (1.91D); for the negative habitual SA group, a smaller DoF was measured with positive AO-induced SA (1.81D) than that with negative AO-induced SA (2.09D). The difference in the DoF of individual participants between the induced positive and negative SA groups showed a quadratic relationship with the habitual SA. Subjective DoF tended to be larger when the induced SA in terms of the sign and magnitude was closer to the participant’s habitual SA, suggesting the importance of considering the habitual SA when applying the extended DoF method using optical or surgical procedures.

## INTRODUCTION

The ability to objectively assess and understand the primary spherical aberration (SA) of the eye has improved owing to the development of various ocular wavefront sensing techniques.^1–3^ SA is the only higher-order aberration (HOA) with a significantly positive population mean.^4,5^ Aging causes progressive alterations in ocular optics, which tends to increase the magnitude of HOAs, especially SA. Moreover, myopic and hyperopic corneal refractive surgeries induce positive and negative SAs, respectively.^3,6^ Given that both signs of SA result in multiple focal points formed by rays passing through different pupil areas, SA represents a useful strategy for extending the depth of focus (DoF).^7–10^ Instead of correcting the corneal SA in presbyopic patients, aspheric optical designs of intraocular lenses (IOLs) generating more SAs can provide a continuous focal zone with a small change in point spread function (PSF). Besides conventional spherical IOLs inducing a positive SA,^11^ recent extended DoF (EDoF) IOLs based on complex asphericity modulation increase the DoF because of the progressive shift in local power from far to intermediate distance.^12–17^

Interestingly, previous studies have reported the DoF of human eyes with the induction of SA, and there were significant variations in DoFs among the studies.^9,15,18–23^ As all of these studies were performed with AO correction, this variability cannot be explained by uncorrected residual aberrations. One of the potential reasons for the variability may be the difference in methodology to define the DoF: 1) visibility of the target and 2) degradation of visual acuity (VA) or contrast sensitivity.^24^ The former has been widely used;^18–20,25–29^ however, it has an intrinsic limitation due to the subjective nature of the blur evaluation.^20^ Moreover, the latter is commonly used based on a resolution threshold.^9,15,21,30^ This method may reduce the effect of between-study variability by its objectivity, but variability was still observed within the previous studies.

Within-study variability can be represented by interindividual variability, and the individual visual system can compensate for the optical blur by neural adaptation.^31^ This neural compensation is not only limited to defocus blur^32,33^ but also to optical blur induced by HOAs at a single object distance.^34–37^ Recently, Zapata-Diaz et al.^29^ expanded the scope further with multiple object distances and demonstrated the significant interindividual variability of the DoF due to neural compensation with the same induced HOA patterns on top of the AO correction of their habitual HOAs. We hypothesized that long-term visual experience with the participants’ habitual SA may affect individual DoF when DoF is extended artificially. It is plausible that long-term exposure to habitual SA modifies the neural processing mechanism to compensate for optical blur at different object distances, resulting in different DoFs under the same optical condition. Therefore, the objective of this study was to assess the neural adaptation effect of a wide range of habitual SA on the DoF. To achieve this aim, we induced a fixed magnitude of SA, but with the opposite sign to the eye, using an adaptive optics (AO) visual simulator to assess the DoF based on the through-focus VA measurement.

## METHODS

### Ethics statements

Written informed consent was obtained from all participants, and study approval was obtained from the University of Rochester Research Review Board. This study adhered to the tenets of the Declaration of Helsinki and was conducted in accordance with the United States Health Insurance Portability and Accountability Act.

### Participants

Nine healthy participants (9 eyes in total) participated in this study. All eyes underwent a recent dilated ophthalmological examination, and no pathology was identified in the normal eyes. Participants with spherical equivalent refractions of more than 1.5 diopters (D) or less than – 1.5 D, astigmatism of more than 0.75 D, or distance best corrected VA worse than 20/20 were excluded. All participants were cyclopledged with 1% tropicamide and 2.5% phenylephrine.

### Adaptive optics visual simulator (AOVS)

The AOVS used in this study has been described elsewhere.^38^ Briefly, it comprises a large-stroke deformable mirror (Mirao 52D; Imagine Eyes, Orsay, France),^39^ custom-made Shack–Hartmann wavefront sensor, a Badal optometer to determine the subjective best focus of the eye, an artificial pupil to control the pupil size, and visual display for VA measurements. The AOVS was operated in a closed loop to control participants’ wavefront aberrations in real time (8 Hz). The wavefront-sensing laser beacon was generated using a superluminescent diode with a center wavelength of 840 nm (bandwidth, 40 nm). Head movements were stabilized with a bite bar, and a phoropter was used to compensate for the sphere and cylinder in some participants partially. Then, the AOVS was used to measure and correct participants’ monochromatic wavefront aberrations over a 6-mm pupil to an accuracy of less than 0.06 μm (<λ/14) as the residual root mean square error and to induce two Zernike primary SA conditions ($C_{4}^{0}=+0.5\;\text{and}\;-0.5\;\mu m$). Thus, every participant was tested under identical optical conditions. Through-focus high-contrast VA was measured, and the AO closed-loop control maintained the optical conditions in real time.

### Through-focus VA measurement

**Figure 1:**
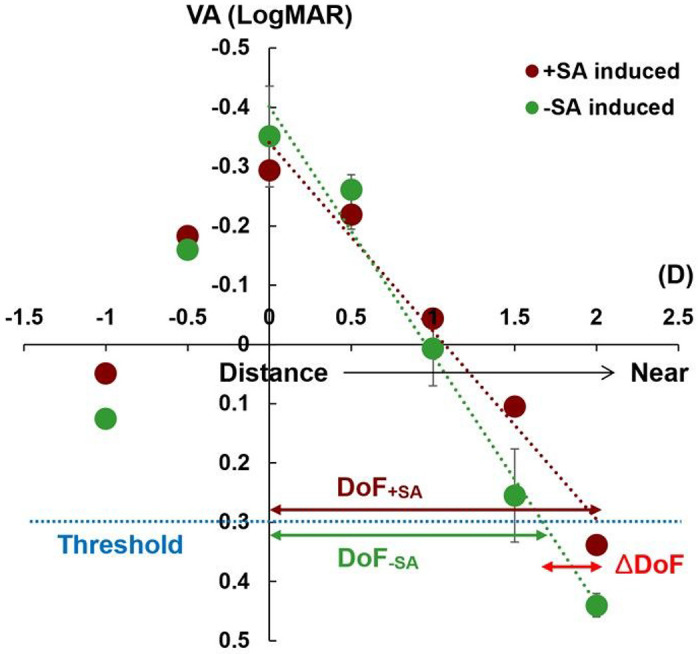
Exemplary through-focus visual acuity (VA) lines from one participant’s eye. The through-focus VA lines were obtained by least-squares linear fitting (R^2^ = 0.97 for the positive adaptive optics [AO]-induced spherical aberration (SA) condition, R^2^ = 0.98 for the negative AO-induced SA condition). The depth of focus (DoF) was defined as the range in diopters (D) from a distance (0 D) to near (positive D) for which acuity surpassed 0.3 logarithm of the minimum angle of resolution or 20/40 on the Snellen acuity scale as a threshold. The difference of DoF (ΔDoF) between the positive AO-induced SA condition (DoF_+SA_) and the negative AO-induced SA condition (DoF_−SA_) was also calculated. Error bars show standard deviations for three repeated measurements.

For each SA condition, the retinal image quality was subjectively optimized to account for the interaction of SA and defocus and to ensure the best image quality at optical infinity, i.e., distance (or far) vision. Through-focus VA was measured at different object distances ranging from − 1 to 2.5 D (0.5-D increments) in a randomized order using a random number table. The high-contrast VA measurement was made with a single tumbling letter “E” (four-alternate forced-choice method) on a uniform background with a mean luminance of 70 cd/m^2^. A narrowband interference filter transmitting 633 ± 5 nm light was used to yield a monochromatic stimulus to remove the chromatic aberration of the eye. A psychometric function based on 30 trials was obtained using the QUEST^40^ algorithm, where acuity was defined as the letter size for which 75% of the responses were correct. Three measurements were averaged for each optical condition and represented in units of the logarithm of the minimum angle of resolution (logMAR). The through-focus VA lines were obtained by least-squares linear fitting using MATLAB (MathWorks, Natick, MA, USA), which only included VA responses from the distance to near defocus range (Fig. 1). Data at the defocus range beyond infinity (negative D) were used to ensure the best visual acuity at 0 D but were excluded from the fitting process. From the through-focus VA curve, the DoF was defined as the defocus range in D from distance (0.0 D) to near (positive D), for which acuity is better than 0.3 logMAR or 20/40 on the Snellen acuity scale.^41^ We also calculated the difference in the DoF between positive versus negative AO-induced SA (ΔDoF = DoF_+SA_–DoF_−SA_).

### Statistical analysis

Data are presented as medians and interquartile ranges (IQRs). Differences in continuous variables (i.e., DoF) between two categorical independent groups (two AO-induced SA conditions [between-participants factor] and two habitual SA subgroups [within-participants factor]) were compared in a non-parametric mixed-effects model using the nparLD R package published by Noguchi et al.^42^ The *post hoc* test, Dunn Q-test (for pairwise comparisons), and Mann–Whitney U-test (for non-pairwise comparisons) were used to analyze data for each pair of subgroups, and multiple comparison errors were corrected using the Bonferroni correction. Statistical significance was set at *P*-values < 0.05. All analyses, including quadratic regression analysis, were performed using RStudio software (Rstudio Team, Boston, MA, USA).

## RESULTS

There were 6 participants with positive SA (age range: 42–57 years, SA range: 0.17–0.8 μm for a 6-mm pupil) and 3 participants with negative SA (age range: 39–59 years, SA range: −1.2 to −0.12 μm for a 6-mm pupil). One participant in the positive SA group underwent myopic corneal refractive surgery, and 2 participants in the negative SA group underwent hyperopic corneal refractive surgery. All 3 participants underwent corneal refractive surgery several years before enrollment (range: 2–4 years). Table 1 summarizes the habitual SAs and DoF values measured under both the positive and negative AO-induced SA conditions for all participants. Generally, a larger DoF was observed in the positive AO-induced SA condition (2.14 D, IQR: 0.43 D) than in the negative AO-induced SA condition (1.96 D, IQR: 0.23 D), but without statistical significance (*P* = 0.354). Additionally, there was no statistically significant difference in the DoF between the positive (2.00 D, IQR: 0.40 D) and negative (2.01 D, IQR: 0.36 D) habitual SA subgroups (*P* = 0.779). There was also no statistically significant interaction between the AO-induced SA groups and habitual SA subgroups (*P* = 0.387).

**Figure 2:**
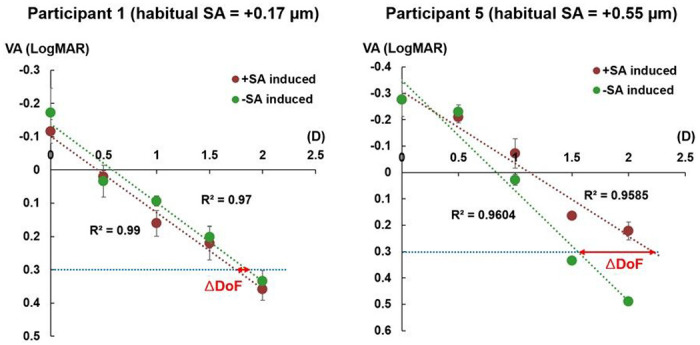
Through-focus visual acuity (VA) lines with different magnitudes of positive habitual spherical aberration (SA). Participant 1 had the most distinct magnitude of habitual SA, while participant 5 had the most comparable magnitude to that of the adaptive optics-induced SA. The through-focus VA lines only included VA responses from the distance to near defocus range. D, diopters; DoF, depth of focus.

Within the positive habitual SA subgroup, a larger median DoF was observed with a positive AO-induced SA condition (2.18 D, IQR: 0.19 D) than with a negative AO-induced SA condition (1.91 D, IQR: 0.25 D), but the difference was not statistically significant (*P* = 0.075). This finding might be due to the wide distribution of habitual SA, averaging the effect of the habitual SA on the DoF. Figure 2 shows two extreme examples of through-focus VA lines with different magnitudes of positive habitual SA from 2 participants. In participant 5, with the closest magnitude of habitual SA (+ 0.55 μm) to that of AO-induced SA (+ 0.5 μm), a larger ΔDoF (0.67 D) was observed compared to participant 1 (−0.11 D), who had the largest difference in magnitude of habitual SA (+ 0.17 μm) compared to that of AO-induced SA.

**Figure 3:**
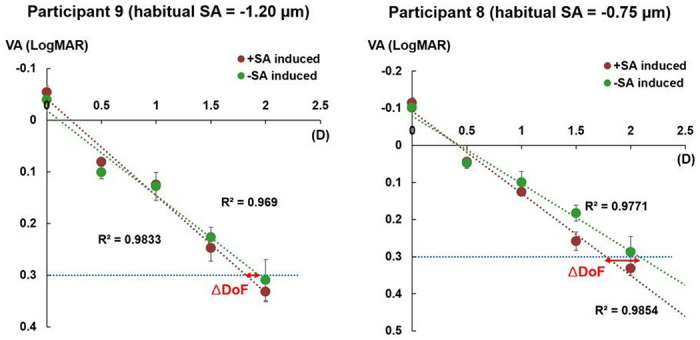
Through-focus visual acuity (VA) lines with different magnitudes of negative habitual spherical aberration (SA). Participant 9 had the most distinct magnitude of habitual SA, while participant 8 had the most comparable magnitude to that of the adaptive optics-induced SA. The through-focus VA lines only included VA responses from the distance to near defocus range. D, diopters; DoF, depth of focus.

Similarly, within the negative habitual SA subgroup, a larger mean DoF was observed with negative AO-induced SA (2.09 D, IQR: 0.23 D) compared to positive AO-induced SA (1.81 D, IQR: 0.23 D), without statistical significance (*p* = 0.275). Figure 3 shows the through-focus VA curves with extremely different magnitudes of negative habitual SA for 2 participants. In participant 8, with the closest magnitude of habitual SA (−0.75 μm) to that of AO-induced SA (−0.5 μm), a smaller ΔDoF (−0.31 D) was observed compared to participant 9 (−0.13 D), who had the largest difference between the magnitude of habitual SA (−1.20 μm) from that of AO-induced SA.

With the positive AO-induced SA condition, there was no statistically significant difference in the DoF between the positive (2.18 D, IQR: 0.19 D) and negative (1.81 D, IQR: 0.23 D) habitual SA subgroups (*P* = 0.606). With the negative AO-induced SA condition, there was also no statistically significant difference in the DoF between the positive (1.91 D, IQR: 0.25 D) and negative (2.09 D, IQR: 0.23 D) habitual SA subgroups (*P* = 0.197).

**Figure 4:**
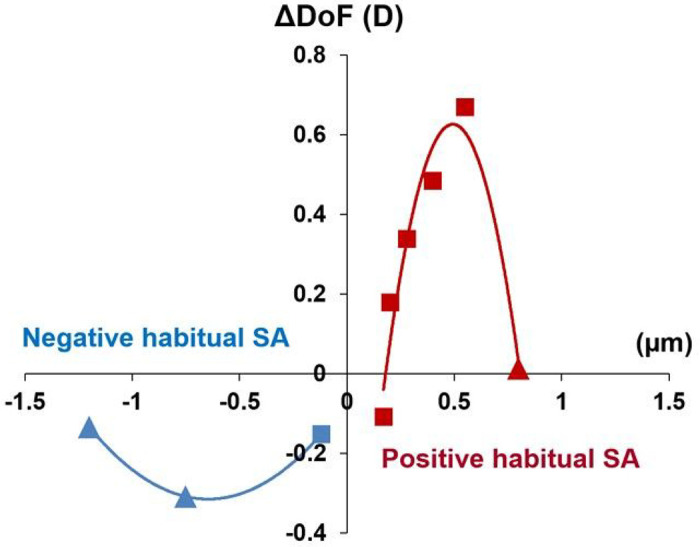
Distribution of the difference in the depth of focus (ΔDoF) with positive and negative adaptive optics (AO)-induced spherical aberrations (SAs). Triangle symbols: participants with corneal refractive surgery; square symbols: participants with no surgery. D: diopters.

Figure 4 shows the distribution of ΔDoF with regard to each participant’s habitual SA. Interestingly, when the regression model was applied, the positive SA subgroup showed a quadratic distribution as a function of habitual SAs (*P* = 0.035, R^2^ = 0.94). In other words, this difference in the DoF tended to be significantly higher when the magnitude of the participant’s habitual SA was closer to that of the positive-induced SA. The DoF in the positive habitual SA subgroup (DoF_+ SA_) was represented by the function defined in equation (Eq.) (1) with respect to habitual SA (SA_nat_) as follows:

1 $$\Delta DoF_{+SA}=-6.38\times {\left(SA_{nat}-0.49\right)}^{2}+0.63\left(D\right)$$

The same trend was observed in the negative SA subgroup, which also exhibited a quadratic distribution. The negative SA subgroup showed the opposite sign in the quadratic regression because we defined the DoF as the DoF with positive AO-induced SA minus the DoF with negative AO-induced SA. A negative DoF indicates that the DoF with a negative AO-induced SA was larger than that with a positive AO-induced SA. The DoF in the negative SA subgroup (ΔDoF_−SA_) was represented by the function defined in Eq. (2), as follows:

2 $$\Delta DoF_{-SA}=0.59\times {\left(SA_{nat}+0.65\right)}^{2}+0.31\left(D\right)$$

## DISCUSSION

The main objective of this study was to investigate whether habitual SA affects the DoF extended by artificially induced SA. Our results showed that while all optical conditions were identical in all participants with AO-based correction and SA induction, the subjective DoFs differed among individuals. Interestingly, the difference in the DoF between positive and negative AO-induced SA tended to be higher when the magnitude of the participants’ habitual SA was closer to that of the AO-induced SA. These results suggest that the individual visual system that adapted to the specific sign and magnitude of the habitual SA plays an important role in determining the subjective DoF.

The balance between the retinal image quality and DoF is essential in the design of various optical treatments for presbyopia, such as contact lenses or IOLs. These optical treatments aim to support the presbyope with optimal retinal image quality over a broad range of object distances. Induction of HOAs, rather than correcting them, has been used to extend the through-focus optical quality of the eye. Rotationally asymmetric aberrations, such as coma-like aberrations, can increase the DoF owing to the increased size of the PSF at the expense of reduced retinal image quality.^9,43–45^ However, there is insufficient consensus on their effect on the estimated DoF. For instance, Rocha et al.^9^ reported that trefoil and coma had no significant impact on the subjective DoF, whereas Legras et al.^43^ demonstrated that coma had a significant influence on the DoF. Moreover, asymmetric aberrations themselves significantly negatively influence retinal image quality, which could be the major reason coma or secondary astigmatic aberrations have drawn less attention. Asymmetric aberrations degrade retinal image quality for far vision, but can increase the DoF of the eye,^46–48^ thus a trade-off exists between an optimal level of retinal image quality and the DoF.^28,49^

**Figure 5:**
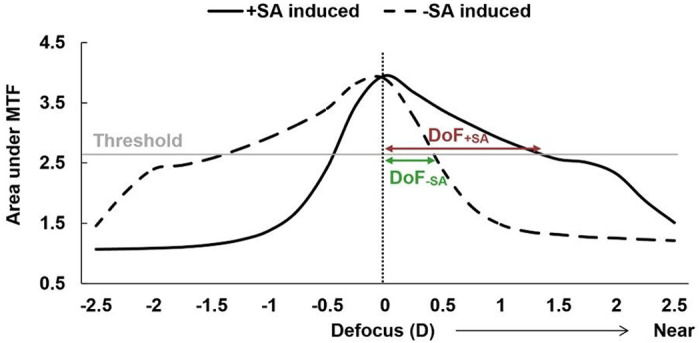
Theoretical estimation of the depth of focus with the positive adaptive optics (AO)-induced spherical aberration (SA) condition (DoF_+SA_) versus the negative AO-induced SA condition (DoF_−SA_). D: diopters, MTF: modulation transfer function.

On the other hand, a radially symmetric aberration, SA increases the DoF with less compromise of retinal image quality at optimal focus.^7,50^ As aforementioned, SA can extend the DoF of the eye, as it can generate multiple focal points near the retinal plane by rays passing through different pupil areas as a function of the radial distance from the center of the pupil. Figure 5 shows the defocus curves of the positive and negative 0.5 μm SAs used in this study. The y-axis shows the area under the modulation transfer function (MTF), and the DoF can be estimated at the threshold used in our study, 0.3 logMAR, by using the area under the MTF, based on a regression model in previous studies.^51,52^ The peaks of the through-focus curves shifted by the induced SAs were corrected to ensure that the retinal image quality was optimal at a far distance (0 D), in accordance with our experimental protocol. Based on the DoF definition in this study, as the defocus ranges from a distance (with the best image quality) to near, a larger DoF was observed with a positive-induced SA condition compared to a negative-induced SA condition in the simulation. This theoretical prediction of optical DoF supports our subjective DoF measured from participants with positive habitual SA. However, the result that the negative habitual SA group showed no statistically significant difference in DoF between the positive and negative induced SA cannot be explained by the simulation. This discrepancy in addition to the observed variation in individual subjective DoF under the same SA conditions, indicates that the optical effect is not the sole factor affecting DoF.

AO has been widely used to separate neural factors from optical factors by creating identical optical conditions for individual subjects by correcting and inducing a certain aberration profile to the eye. Atchison et al.^27^ found considerable interindividual variability in noticeable and objectionable blur, even though they corrected all HOAs in their participants, indicating that the neural processing associated with blur detection might vary among participants. Zapata-Diaz et al.^29^ paid attention to this interindividual variability and assessed whether habitual HOA alone or neural factors can explain this variability. By swapping participants’ habitual HOAs with each other, they observed that inducing new HOA patterns tended to reduce the DoF in most participants and that the effect of neural adaptation on the interindividual variability of DoF with the new optics was significantly greater than that with their habitual HOAs. Previous studies^9,21^ have reported the inter-subject variability of the DoF with the identical optical condition (AO-induced SA + habitual aberration correction) among individuals; however, the potential correlation between the finding and habitual SA has not been studied. A recent study^15^ presented the interindividual variability of the DoF with the same optical condition (SA-based EDoF IOL profile + habitual aberration correction); the authors explained that they could not find a tendency associated with the magnitude of the habitual SAs, but the magnitude of their habitual SAs was small. These previous studies involved much narrower ranges of participants’ habitual SA (ranges [μm]: −0.07–0.23^9^ and – 0.13–0.38^21^ with a 6-mm pupil and 0–0.14^15^ with a 4.5-mm pupil) compared to ours (range: −1.20–0.80 μm with a 6-mm pupil).

How does habitual SA affect the DoF induced by SA? Contrast perception by the human visual system is established with multiple spatial frequency-specific channels in the visual cortex.^53^ These channels can adjust contrast gains in response to changes in the visual environment. Georgeson and Sullivan^54^ proposed that a continuous feedback mechanism in the visual system could compensate for the attenuated contrast by the ocular optics to maintain perceived contrast constant across a wide spatial frequency range as long as retinal contrast is above the threshold, i.e., “contrast constancy.”^33^ Furthermore, the human neural system is capable of compensating for optical blur induced by defocus.^32,33^ Subsequent studies have reported that the neural system of healthy eyes, exhibiting typical optical quality,^34,55^ as well as those with severe optical abnormalities,^35,36^ can also compensate for habitual optical blur induced by HOAs after both short-^37^ and long-term exposure^36,56^ to aberrated stimuli. We have recently proposed an additional neural compensation mechanism, the partial restoration of phase congruency by altering phase perception for habitual optical blur.^57,58^ These neural compensatory mechanisms may explain our extended findings involving adjusting contrast gain and phase relationships of various spatial frequencies. Adaptation might occur based on the entire aberration pattern (or blur) rather than individual aberration modes, each having distinct impacts on contrast and phase shift at different spatial frequencies. In the context of the present study, SA could be the aberration that contributes most to adaptation, given its magnitude is much larger than other HOAs. This does not negate the potential contribution of other HOAs, but we speculate that their impact would be relatively smaller than SA, especially in individuals with large habitual SA. Moreover, prolonged visual experience with a certain sign and magnitude of habitual SA combined with various magnitudes of defocus (i.e., from far to near vision) may allow the neural system to compensate for blur under multiple optical states.^59^ This provides important insights into the capacity of the presbyope’s neural system to deblur not only for a single distance but also for a range of target vergences.

In Fig. 4, the positive and negative habitual SA groups showed different quadratic fit in terms of the maximum absolute ΔDoF and shapes. This result may be due to interindividual differences in the exposure period to chronic blur. Participants who underwent corneal refractive surgery had much shorter adaptation periods (PS6: 4 years, PS8: 2 years, and PS9: 3 years) than those with normal optics. In some of these participants, the neural adaptation process may still be in progress. This might mean that a larger blur compensation could be gained later as they experience their “habitual” SA longer, resulting in a larger DoF, especially for the negative habitual SA group, where three of the four participants underwent corneal refractive surgery. However, there is clinical evidence regarding the timescale for neural adaptation to enhanced optical quality following corneal refractive surgery. Pesudovs^60^ focused on visual acuity changes over shorter time intervals post-LASIK, reporting that the neural system typically requires approximately 10 weeks to adapt to a new state of blur following refractive surgery. This duration is notably shorter than the adaptation periods covered in our study. Further investigations are required to support this explanation.

These findings have important clinical implications. Presbyopia-correcting IOLs have been designed to have different SAs, and individual patients also have diverse magnitudes of corneal SA; therefore, the consequent total SA after cataract surgery will vary in terms of the sign and magnitude. This variation in the ocular SA could affect the subjective DoF, even if some IOLs are not specifically designed to extend the DoF with SA modulation. The neural compensatory mechanism evoked by individual habitual SA could also affect the subjective DoF, at least in the short term after surgery. Moreover, given that neural plasticity seems to exist in aged pseudophakic eyes,^61^ the aging visual system may be capable of re-adapting to new optics (i.e., IOLs) over time, resulting in increasing DoF. It would be intriguing to examine the extent to which plasticity improves subjective DoF with presbyopia-correcting optical/surgical treatments using a longitudinal observational study design.

A major limitation of our study was the small sample size (n = 9). Since we sought to recruit participants with a wide range of habitual SAs, obtaining a larger sample size for statistical power was challenging. Finding participants with a substantial magnitude of SAs and no history of corneal refractive surgeries also proved challenging. According to the sample size calculation derived from our results, a minimum of 15 participants for each habitual SA group is necessary for the future study. Furthermore, if chromatic stimuli resembling natural scenes were employed, the subjective DoF might differ due to the presence of longitudinal chromatic aberration. On the other hand, the impact of the Stiles – Crawford effect (SCE) combined with SA on DoF was not considered in our study. It has been reported that both the SCE and SA increased DoF, and the SCE improved image quality in an aberrated eye with SA only when defocus and SA have the same sign.^62^ Another study reported that the SCE had a small compensating capability for SA (and defocus).^63^ Moreover, it is crucial to note that the influence of the SCE on DoF with higher magnitudes of SA in our study (0.5 μm in 6 mm pupil) could differ from that in previous literature (0.2 μm in 6 mm pupil), and there might be interindividual variation in the SCE. In addition, although participants had a practice session to familiarize themselves with the experimental tasks before the data collection, there might have been short-term adaptation to artificially induced blur that might have affected the subjective DoF.^64^ However, short-term blur adaptation was also reported to be limited after the first 6 minutes;^65^ therefore, this effect would have been insignificant after the practice session in our study. Herein, we used AO to correct all monochromatic aberrations and examined the visual response to induced SA to determine whether habitual SA can influence the subjective DoF. Our findings suggested that neural adaptation to habitual SA compensated for the optical blur from far to near vision perceptually, resulting in extending the DoF. Therefore, the outcomes of optical treatments for presbyopia could vary due to the neural compensatory mechanism based on their habitual optics.

## Figures and Tables

**Table 1:** Summary of the depth of focus (DoF) measurements in both positive and negative adaptive optics (AO)-induced spherical aberrations (SAs) with a broad spectrum of habitual SAs.

Participant	Habitual SA (μm)	DoF (D)		Difference (ΔDoF, D)
		+SA induced	−SA induced	
P1	0.17	1.75	1.86	−0.11
P2	0.20	2.14	1.96	0.18
P3	0.28	2.02	1.68	0.34
P4	0.40	2.46	1.98	0.48
P5	0.55	2.23	1.56	0.67
PS6	0.80	2.24	2.23	0.01
P7	−0.12	2.25	2.40	−0.15
PS8	−0.75	1.78	2.09	−0.31
PS9	−1.20	1.81	1.94	−0.13

Data are averages of three measurements.

D, diopters; P, participant; PS, participant with corneal refractive surgery.

## Data Availability

The datasets used and/or analysed during the current study are available from the corresponding author on reasonable request.
